# End-of-life decision making by Austrian physicians - a cross-sectional study

**DOI:** 10.1186/s12904-019-0509-3

**Published:** 2020-01-04

**Authors:** D. Jahn-Kuch, A. Domke, S. Bitsche, H. Stöger, A. Avian, K. Jeitler, N. Posch, A. Siebenhofer

**Affiliations:** 1 Palliative Care Unit, Department of Internal Medicine, Medical University of Graz, Graz, Austria; 2 Institute of General Practice and Evidence-based Health Services Research, Medical University of Graz, Graz, Austria; 3 Institute for Medical Informatics, Statistics and Documentation, Medical University of Graz, Graz, Austria; 4 Institute of General Practice, Goethe-University Frankfurt am Main, Frankfurt am Main, Germany

**Keywords:** End-of-life decisions, Quantitative research, Palliative care, Physician-assisted suicide

## Abstract

**Background:**

Austria has recently been embroiled in the complex debate on the legalization of measures to end life prematurely. Empirical data on end-of-life decisions made by Austrian physicians barely exists. This study is the first in Austria aimed at finding out how physicians generally approach and make end-of-life therapy decisions.

**Methods:**

The European end-of-life decisions (EURELD) questionnaire, translated and adapted by Schildmann et al., was used to conduct this cross-sectional postal survey. Questions on palliative care training, legal issues, and use of and satisfaction with palliative care were added. All Austrian specialists in hematology and oncology, a representative sample of doctors specialized in internal medicine, and a sample of general practitioners, were invited to participate in this anonymous postal survey.

**Results:**

Five hundred forty-eight questionnaires (response rate: 10.4%) were evaluated. 88.3% of participants had treated a patient who had died in the previous 12 months. 23% of respondents had an additional qualification in palliative medicine. The cause of death in 53.1% of patients was cancer, and 44.8% died at home. In 86.3% of cases, pain relief and / or symptom relief had been intensified. Further treatment had been withheld by 60.0%, and an existing treatment discontinued by 49.1% of respondents. In 5 cases, the respondents had prescribed, provided or administered a drug which had resulted in death. 51.3% of physicians said they would never carry out physician-assisted suicide (PAS), while 30.3% could imagine doing so under certain conditions. 38.5% of respondents supported the current prohibition of PAS, 23.9% opposed it, and 33.2% were undecided. 52.4% of physicians felt the legal situation with respect to measures to end life prematurely was ambiguous. An additional qualification in palliative medicine had no influence on measures taken, or attitudes towards PAS.

**Conclusions:**

The majority of doctors perform symptom control in terminally ill patients. PAS is frequently requested but rarely carried out. Attending physicians felt the legal situation was ambiguous. Physicians should therefore receive training in current legislation relating to end-of-life choices and medical decisions. The data collected in this survey will help political decision-makers provide the necessary legal framework for end-of-life medical care.

## Background

In recent years, debates concerning ethical and legal issues surrounding the care of patients at the end of life have intensified, both in- and outside Austria, and have aroused public interest [1–3]. A fear of intolerable suffering, a loss of control over one’s own being, and dependence on care provided by others, are some of the reasons why an increasing number of patients worldwide wish their lives to be ended prematurely via physician-assisted suicide (PAS), or “killing on request” (KoR) [4–7].

Throughout the world, there are clear differences in physicians’ attitudes towards taking measures to end lives prematurely. However, physicians in countries in which PAS and/or KoR are no longer punishable by law generally have a much more positive attitude towards the use of such measures than those in countries in which they are illegal [8–12]. In the current Austrian criminal justice system, life is an inalienable and legally protected right. Even with the express will of the person concerned, providing any help to end someone’s life, as well as active assistance to commit suicide is punishable by law. This is regulated in the following paragraphs of the Criminal Code (Federal Chancellery 2016b):“Killing on demand” § 77. Whoever kills another person upon their serious and sincere request will be subject to between 6 months and 5 years imprisonment. “Cooperation in suicide” § 78. Whoever encourages another person to kill himself or helps him to do so will be subject to between 6 months and 5 years imprisonment. However, according to the Austrian legal system, patients have the right to refuse medical treatment, even if this causes premature death. But this presupposes that the respective person is capable of discernment and judgment. Medical treatment against the will of a patient is not admissible (§ 110 StGB, unauthorized treatment). Although PAS is prohibited by Austrian law, and this stance is supported by the National Medical Association, it should at least be discussed by the National Bioethics Commission [13, 14].

This study is the first in Austria that has set itself the objective of finding out how physicians generally approach and make end-of-life therapy decisions, what their views are on measures to end lives prematurely, as well as the current legal situation and legal training.

## Methods

### Design

Quantitative cross-sectional study.

### Setting

The survey was aimed at physicians that, as a result of their specialization, provide end-of-life care to their patients particularly frequently. Participants were therefore specialists in internal medicine with hematology and medical oncology as a sub-specialty, specialists in internal medicine with or without a sub-specialty, and general practitioners working in private practice (GPs).

### Sampling and recruitment

**Fig. 1:**
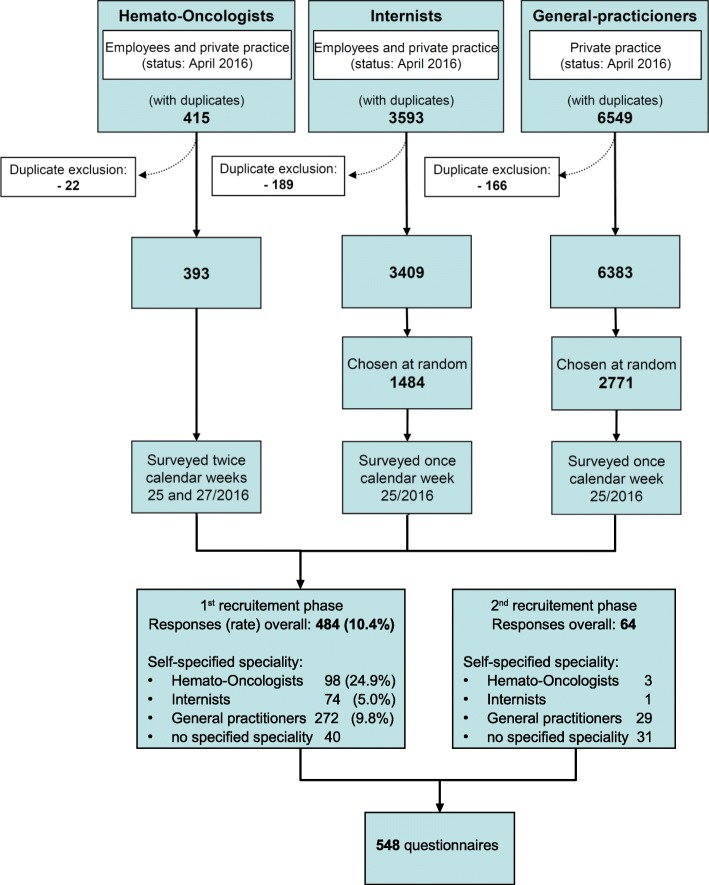
Selection of participants and response rate. Flow chart of recruited hemato-oncologists, internists and general practicioners for the questionnaire * 1 HO=Hemato-oncologists, I=Internists, GP = General practitioners, n.s. = not specified

All specialists in internal medicine whose sub-specialty was hematology and medical oncology and who worked in Austria were surveyed (*n* = 393). Additionally, a random sample survey of all specialists in internal medicine working either in private practice or as salaried employees in Austria that did not belong to the group mentioned above, as well as all GPs in private practice, was performed once. Overall, 43.4% of both the specialists in internal medicine and the GPs were surveyed (specialists in internal medicine *n* = 1484, GPs *n* = 2771) (Fig. 1). As the initial response rate was unsatisfactory (June 20 to November 23, 2016), a second recruitment phase was initiated (November 24 to March 2, 2017). The second phase involved sending the questionnaire to all specialists employed at the Clinic for Internal Medicine of the LKH State University Hospital in Graz, and promoting the online procedure at an Austrian General Practice Congress in November 2016 [15].

### Questionnaire and data collection

There were two parts to the survey. In the first part, the approved and slightly modified version of the European end-of-life decisions (EURELD) consortium questionnaire translated by Schildmann et al. was used [2, 16, 17]. This questionnaire specifically asked doctors that had provided care to patients that had died during end-of-life treatment in the previous 12 months about the measures they had used, withheld, or intensified. Some explanations to the questions were adapted to take into account the legal situation in Austria. In the second part, which consisted of seven further items, the physicians were asked about palliative medicine, training, and the legal situation in Austria (legal certainty). These seven items were pilot tested in a survey of seven doctors. The comprehensibility of the items was clearly confirmed by those participants. In an open question, the physicians were also asked for improvement suggestions relating to palliative care and palliative care training. A total of 76 responses were submitted and underwent free-text analysis. The answers were ordered according to thematic context and categorized into 9 groups. The physicians were further asked to provide demographic information on themselves and their patients. Upon request, the questionnaire is available from the authors.

The physicians were invited to participate in the survey by letter. They could either complete a paper and pencil version of the questionnaire and send it back, or answer the questions online using the SurveyMonkey webapp. As neither the questionnaires nor the link to the online survey were personalized, data collection was completely anonymous.

### Analysis

Items were analyzed in terms of absolute numbers and percentages. Group comparisons (e.g. internal medicine physicians vs GPs vs hemato-oncologists; age groups) were conducted using the chi-square test or Fishers’s exact test, as appropriate. Responses to open-ended items were grouped according to content and analyzed descriptively. Questionnaires with missing data were also included in the analysis, but missing data were not imputed. For all analyses, *p* < 0.05 was considered statistically significant. SPSS 24.0.0.0 (IBM Corp., Armonk, USA) was used in data analysis.

### Ethical approval

Upon written request, the chairman of the local ethics committee decided approval was unnecessary as no patients were to be involved in the survey (statement 15.3.2016). It was an anonymous questionnaire; therefore, no consent from the participating doctors was required.

## Results

After completion of the two recruitment phases, 548 questionnaires were available for analysis, of which 484 came from the first recruitment phase (response rate 10.4%) and 64 from the second (see Fig. 1). As the size of the study population in the second recruitment phase was unknown, it was not possible to calculate the response rate. Unless otherwise stated, all percentages are based on the entire surveyed population.

### Participant characteristics

36.9% of the physicians (*n* = 202) were female. The 56–65 year age group was the most strongly represented (34.1%). Four hundred seventy-seven physicians could be clearly allocated to a particular specialty, of whom 101 (18.4%) were hemato-oncologists, 75 internal medicine physicians with or without an additional sub-specialty, and 301 (54.9%) GPs. 126 (23%) of the 548 said they had an additional qualification in palliative medicine. 63.0% (*n* = 345) of the respondents were catholic and 6.6% (*n* = 36) protestant (See Table 1). 35.1% (*n* = 194) said their religious faith played a role in their decision-making, while 55.3% (*n* = 303) said this was not the case.

**Table 1:** Characteristics of answering physicians (*n* = 548)

	Number	Percent
Sex		
Female	202	36.9
Male	302	55.1
not specified	44	8.0
Age		
under 36 years	29	5.3
36–45 years	110	20.1
46–55 years	161	29.4
56–65 years	187	34.1
over 65 years	18	3.3
not specified	43	7.8
Religion		
Catholic	345	63.0
no religion	111	20.3
not specified	47	8.6
Protestant	36	6.6
another religion	9	1.6
Physician’s specialty		
General practitioner	301	54.9
Hemato-oncologist	101	18.4
Internal medicine physician	75	13.7
Not specified	66	12.0
Not included	5	1.0
Additional qualification		
Additional qualification in palliative medicine (Diploma from Austrian Medical Chamber)	126	23.0

### Patient characteristics

484 (88.3%) of the 548 physicians said they had provided healthcare to a patient that had died during the course of treatment during the previous 12 months. In 430 cases, the patient had not died suddenly or unexpectedly. The median age of the patients (*n* = 484) was 78 years. 52.3% (*n* = 253) of the patients were male. Of the 484 patients, 217 (44.8%) died at home and 183 (37.8%) in hospital. Cancer diseases were responsible for the clear majority of deaths (53.1%, *n* = 263), followed by cardiovascular diseases (22.2%, *n* = 110) (Table 2).

**Table 2:** Characteristics of patients that had died during the course of treatment during the previous 12 months (*n* = 484)

	Number	Percent
Gender		
Male	253	52.3
Female	223	46.1
Not specified	8	1.7
Place of death		
Home	217	44.8
Hospital	183	37.8
Old people’s home, nursing home, care home	62	12.8
Not specified	13	2.7
Elsewhere	9	1.9
Cause of death (Multiple answers possible)		
Cancer	263	53.1
Cardiovascular disease	110	22.2
Neurological disease (incl. stroke)	27	5.5
Other or unknown	54	10.9
Respiratory tract disease	24	4.8
Not specified	17	3.4
Age, median (min – max)	78 (19–101) years	

### Physicians’ treatments at end of life

In the 430 patients that did not die suddenly or unexpectedly, 86.3% of the doctors said they had intensified medical treatments to relieve pain and/or symptoms. 60% withheld further treatment, such as the administration of antibiotics or blood products, and 49.1% discontinued a treatment. 63.5% of 219 responding physicians assumed that their decision was unlikely to have shortened the patient’s life at all, or by less than 24 h. 20.1% estimated that the patient’s life was shortened by 1–7 days.

Five doctors (1.2%) said they had prescribed, made available or administered a medication with the intention of hastening death, or providing the patient with a means to end his or her life. In two cases, the patient took the medication. In two cases, the physician administered it upon the expressed request of the patient, and on one occasion it was administered by the physician and a nurse although the patient had expressed no such wish. An organization providing assisted suicide was not involved in any of the five cases (Table 3).

**Table 3:** Actions carried out at the end of the life of a patient that did not die suddenly or unexpectedly (*n* = 430)

	Number	Percent
Did you do, or arrange that someone else did, the following? (Multiple answers possible)		
Intensify a medical treatment to relieve pain and/or symptoms	371	86.3
Decide to withhold a treatment	258	60.0
Discontinue a treatment	211	49.1
Did the patient receive medications such as barbiturates or benzodiazepines to maintain continuous deep sedation or a coma until death?	72	16.7
Did death result from a medication that you prescribed, made available or administered with the express intention of hastening death (or enabling the patient to end his or her own life)?	5	1.2

The possible consequence of taking measures to shorten a patient’s life was discussed with the patient by 47.2% (*n* = 100) of the doctors that answered this question. The most common reason for not doing so (32.1%) was that it was obviously in the best interest of the patient (*n* = 36).

78.1% (*n* = 428) of the doctors had on occasion asked palliative care services to help care for the patient. 89.7% (*n* = 384) felt that the services were helpful. Hemato-oncologists (89.9%, n = 89) and GPs (84.2%, *n* = 251) were more likely to avail themselves of mobile palliative care services than internal medicine physicians to a statistically significant degree (62.5%, *n* = 45; *p* < 0.001). Although 71.7% (*n* = 393) of respondents said they had been adequately trained to deal with end-of-life patients, only 45.1% (*n* = 247) felt they were legally secure when treating such patients.

### Attitudes towards physician-assisted suicide

41.4% (*n* = 227) of the surveyed physicians said they had been asked to provide assisted suicide in the past. 51.3% (*n* = 281) of the respondents said that assisted suicide was completely out of the question, while 30.3% (*n* = 166) would only consider it under certain conditions. 14.2% (*n* = 78) said they were undecided. 38.5% (*n* = 211) of the physicians were in favor of the current prohibition of PAS suicide in Austria, while 23.9% (*n* = 131) were against it, and 33.2% (*n* = 182) undecided. Younger doctors were more likely to consider helping a patient commit suicide under certain conditions than older doctors (under 36 years: 55.2%; over 65 years: 22.2%; *p* = 0.026).

Respondents that had already been asked to provide PAS (39.1%, *n* = 89) were, under certain conditions, considerably more likely to support a patient that wanted to commit suicide than those that had not (26%, *n* = 77; *p* = 0.001). This group was also statistically significantly more likely to be against the prohibition of PAS (30.4%, *n* = 69) than those that had never been asked (20.9%, *n* = 62; *p* = 0.047).

### Sub-group analyses

#### Influence of medical specialty

A comparison of the responses of individual groups according to medical specialty revealed statistically significant differences: hemato-oncologists indicated that they had decided to withhold treatments, discontinue therapies, and intensify treatment with medications to relieve pain and symptoms, far more frequently than GPs and internal medicine physicians. Furthermore, hemato-oncologists were far more likely to use benzodiapines and barbiturates for the deep sedation of patients, were considerably more likely to regard themselves as adequately trained to provide end-of-life care, viewed themselves as legally more secure, and were more often asked to provide PAS (see Table 4 for further details).

**Table 4:** Sub-group analyses according to professional qualification

	Hemato-oncologists	Internal medicine physicians	GPs	Overall	*p*-value
Intensification of medical treatment to relieve pain and/or symptoms	95.8% (91)	79.6% (39)	86.6% (201)	88% (331)	*p* = 0.01
Decision to withhold a treatment	76.6% (72)	53.1% (26)	56.6% (133)	61.1% (231)	*p* = 0.002
Discontinuation of a treatment	61.5% (56)	57.1% (28)	44.2% (103	50.1% (187)	*p* = 0.011
Administered benzodiazepines or barbiturates for deep sedation	31.9% (29)	16.7% (8)	10.3% (23)	16.5% (60)	*p* < 0.001
Do you feel adequately trained to provide patients with end-of-life care?	91.8% (90)	78.6% (55)	73.3% (214)	78% (359)	*p* = 0.001
I feel legally secure when providing patients with end-of-life care	61% (61)	41.1% (30)	43.4% (128)	46.8% (219)	*p* = 0.005
Have you been asked to provide physician-assisted suicide in the past?	66% (66)	35.1% (26)	39.9% (119)	44.7% (211)	*p* < 0.001

#### Influence of the physician’s age

A greater proportion of younger physicians felt that under certain circumstances, it would be appropriate to help a patient commit suicide than older physicians. While this was the case for 55.2% (n = 16) of those under 36 years of age, it was only the case for 25.7% (n = 28) of those aged 36–45, 33.3% (*n* = 53) of those aged 46–55, 31.6% (*n* = 59) of those aged 56–65, and 22.2% (n = 4) of those over 65.

#### Influence of the physician’s experience

Doctors that had already been asked to assist in committing suicide (*n* = 227) were more often able to imagine providing assistance under certain conditions than those (*n* = 296) that had not (39.2% (*n* = 89) vs. 26.0% (*n* = 77)). The difference was statistically significant.

#### Additional qualification in palliative medicine

126 (23.0%) of the 548 surveyed doctors said they had an additional qualification in palliative medicine. 34.7% (*n* = 35) of the 101 hemato-oncologists, 24.0% (*n* = 18) of the 75 internal medicine physicians and 22.6% (*n* = 68) of the 301 GPs had an additional qualification in palliative medicine. Having an additional qualification in palliative medicine was not statistically significantly associated with the likelihood to intensify treatments to relieve pain and/or symptoms at the end of life (no additional qualification 87.5%, with additional qualification 93.3%; *p* = 0.099). In Austria, having the additional qualification was also not associated with attitudes towards the prohibition of PAS (for/against/undecided: without additional qualification: 38.7% / 25.2% / 36.2%, with additional qualification: 45.5% / 24.4% / 30.1%; *p* = 0.345).

#### Patients that died of cancer vs. other patients

53.1% of all patients involved in this study died of cancer. In patients that died of cancer, treatment was discontinued statistically significantly more frequently (55.3%) than in other patients (42.9%; *p* = 0.049), and medical treatment to relieve pain and/or symptoms was statistically significantly more often intensified (96.0%) than in patients that died of a non-oncological disease (81.9%; *p* < 0.001). Physicians were statistically significantly less likely to assume that death would be hastened by the discontinuation of a treatment in oncological patients (43.4%) than in non-oncological patients (62.2%; *p* = 0.027).

#### Age groups

In the group of patients aged over 80 years, the decision to withhold a treatment was made statistically significantly more often (69.5%) than in patients under 80 years of age (56.8%; *p* = 0.009), but treatment to relieve pain and/or symptoms was less often intensified (patients aged over 80 years: 82.8%, under 80 years: 93.2%; *p* = 0.001).

#### Religiosity

Both for physicians whose religious beliefs play a role in the way they exercise their profession, and those for whom it is not relevant, the percentage in favor of the prohibition of PAS is higher than the percentage against it (religious beliefs play a role: 44.8% vs. 25.0%, religious beliefs do not play a role: 38.2% vs. 23.3%). In terms of percentages, the differences between the two groups were not statistically significant (*p* = 0.157).

### Free-text answers

103 (18.8%) of the physicians took advantage of the opportunity to make free-text comments. The most common recommendation was that palliative care be supported by means of improved remuneration and the increased availability of inpatient palliative and hospice care, particularly in rural areas (*n* = 20). Doctors also mentioned the legal minefield in which physicians treating end-of-life patients find themselves, and called for a debate to take place on the legalization of PAS (*n* = 7). They also saw a need for further training for both doctors and nurses (*n* = 6).

## Discussion

### Main findings

The aim of the study was to gain a first insight into the treatment decisions facing Austrian doctors in the care of end-of-life patients. Five hundred forty-eight questionnaires were available for analysis, and 88.3% of the surveyed physicians had looked after a patient that had died within the previous 12 months. Although almost half of them (47.6%) felt the legal situation was ambiguous, and 21.4% did not feel they had been adequately trained to deal with patients at the end of their lives, 60% had decided to withhold further treatment and 49.1% had discontinued existing treatment. Medical treatment for the relief of pain and/or symptoms had been intensified in 86.3% of cases. In five cases, death had resulted from taking a medicine that had been prescribed, made available or administered by one of the participants. 41.4% of those surveyed had been asked for PAS in the past, with hemato-oncologists being asked statistically significantly more often than other physician groups. PAS is rarely practiced and was out of the question for 51.3% of respondents. 38.5% were in favor of the current prohibition of PAS.

### Attitudes towards ending lives prematurely

The results presented here differ markedly from the survey of German doctors conducted in five Federal states by Schildmann et al. 2014 [2]. Both decisions to withhold and to discontinue treatment occurred more frequently in the Austrian study than in the German one. Furthermore, in the study by Schildmann et al., a lower percentage of doctors rejected the idea of assisting suicide (42%) than in the Austrian study (54%), a higher percentage of physicians had administered medications for continuous sedation until death (31%, Austria: 18%), a lower percentage of physicians had decided to withhold a treatment (51%, Austria: 62%) and a lower percentage had discontinued a therapy (42%, Austria: 51%). The explanation for the differences may be that the present study did not survey doctors from all medical professions but focused on doctors that are relatively frequently confronted with the death of patients. A further reason may be that the legal situation in Austria and Germany differs. Both “killing on request” (KoR) and assisted suicide are prohibited by the national penal code and can result in between 6 months and 5 years imprisonment in Austria. In Germany, KoR is punishable by up to 5 years imprisonment, but suicide is considered to be an expression of self-determination and also exempt from punishment when assisted.

The response rates in the two studies also differed. It can therefore not be ruled out that selection bias was responsible for the differences in the answers.

PAS is prohibited in Austria. 40% of surveyed doctors were in favor of adhering to current laws, while 25% were against the prohibition of PAS. In a survey of the attitudes of 4000 doctors towards the legalization of PAS, a substantially higher proportion of doctors were in favor of legalizing it in both the U.S. and Europe (USA: 54%, Germany and Great Britain:47%, Italy: 42%, France: 40%) than in the present study [18]. However, the possible consequence of taking measures to shorten a patient’s life was discussed with the patient by less than 50% of the doctors in this study. By neglecting the patient in decisions concerning medical issues at the end of life, doctors showed that they still have a paternalistic understanding of their relationship to patients. From an ethical point of view, this attitude is problematic. On the other hand, it should be borne in mind that the explanatory power of quantitative data in complex processes, such as decision-making at the end of life, is limited. Various qualitative test results (e.g.: Schildmann et al., 2011) showed that oncologists do not always involve patients in the decision-making process. Possible reasons and influencing factors for this behavior should be assessed in further studies.

Although the legal situation surrounding end-of-life care is clear, only 45% of respondents to our investigation felt legally secure in the treatment of patients at the end of life. This clearly shows that physicians should receive training in current legislation relating to end-of-life choices and medical decisions.

Similar to other studies, our survey showed that PAS is regarded considerably more positively by young doctors than by older and more experienced physicians [5, 18, 19].

The question whether the decision to withhold or discontinue a treatment may have shortened the lives of patients was answered very differently in our study than in that of Dahmen et.al., who used the same survey instrument. In the study by Dahmen et al., 19.8% of the respondents reckoned the decision to limit treatment did not shorten the patient’s life, while 40.6% thought it may have been shortened by 1 to 7 days [20]. In our study 63.5% doubted whether the patient’s life had been shortened, with only 20.1% considering it to have been curtailed by 1 to 7 days.

### Influence of an additional qualification in palliative medicine

Numerous international studies have demonstrated that doctors with an additional qualification in palliative medicine are much more likely to reject the use of measures to end lives prematurely than those without [21–24]. However, this observation differs from the results of the present study. Interestingly, an additional qualification in palliative medicine had no statistically significant influence on the likelihood that doctors, under certain circumstances, would assist patients that wished to commit suicide. The additional qualification also had no statistically significant influence on attitudes towards the prohibition of PAS. Statements by the Austrian Palliative Care Association and the European Association for Palliative Care [25, 26], which both firmly reject KoR and PAS, could have been expected to generate a clear majority in favor of prohibition and against support for PAS.

### Influence of religiosity

In a survey of 2000 U.S. physicians from different professional backgrounds, Curlin et al. came to the conclusion that religious doctors are statistically significantly more likely to reject assisted suicide and terminal sedation than their non-religious colleagues [27]. This observation is true for most such investigations. The physician’s religious beliefs are the principal reason for voting against the legalization of KoR [11, 28, 29]. In the present study, however, the percentage of doctors in favor of the prohibition of PAS was similarly large (44.8%) among both doctors for whom religious beliefs were important and those for whom they played no significant role.

### Strengths and limitations

To the best of our knowledge, this is the first major Austrian survey to investigate doctors’ end-of-life therapy decisions, their experiences with PAS and their attitudes towards its prohibition.

A large percentage of surveyed doctors had provided health care to a patient that had died during the previous 12 months. We can therefore assume that end-of-life decision making was common in the surveyed group of doctors.

When interpreting the data, it is important to point out that there are a number of reasons why the results are not representative of all doctors practicing medicine in Austria. On the one hand, the response rate of 10.4% of distributed questionnaires was not only well below our expectations but also well below the response rates that prevailed in comparable investigations [2, 9, 16]. On the other hand, limiting the selection of participants to physicians whose professional backgrounds meant they were likely to provide health care to end-of-life patients does not mean it is possible to conclude that doctors specializing in different fields would have behaved similarly. In addition to the selection of the target group, the validity of the investigation may also have been compromised by inaccuracies in the memory of events that took place months previously.

It appears likely that the low response rate partially reflects the delicate and sensitive nature of the topic and associated concerns about the anonymity of the study. It may also be indicative of the false impression that the aim of the questionnaire was primarily to find out the extent to which assisted suicide had actually taken place. Furthermore, the style of some of the questions was rather technocratic and clear-cut and lacked the human component that is more appropriate to the topic. Despite these limitations, we decided to use the present questionnaire because it is well established in several countries and investigates various dimensions that are relevant to the subject, such as specific therapy decisions, communication and the legal situation.

## Conclusions

In 2014, the Austrian parliament set up a commission to study “Dignity at the end of life”. The result was a position paper containing a total of 51 recommendations, one of which was a call to expand hospice and palliative care programs in Austria [30].

Partially as a result of very detailed free-text answers, the present study reinforces the recommendation of the Study Commission of the Austrian Federal Chancellery [31] that the reach of palliative care in Austria should be extended and that medical, ethical and legal training relating to the treatment of dying patients should be improved.

Almost 50% of the physicians that participated in this survey felt legally insecure when treating end-of-life patients. It appears that many doctors regard the legal consequences of end-of-life medical treatments as ambiguous. These concerns should be taken into consideration in specifically designed training courses. Furthermore, this study should encourage follow-up projects dealing with the implementation of palliative healthcare concepts in in- and outpatient care, as well as nursing institutions. Further research projects should examine existing communication instruments such as patient decrees, health care proxies, advance care planning and the “provision dialogue” developed for nursing homes under the auspices of “Hospice Austria”. These projects should determine the acceptance of such tools in society, as well as their implementability and relevance in everyday life, but also identify possible failings [32].

The low response rate may be seen as an expression of the reluctance of physicians to openly discuss such subjects as dying, death and associated demands for measures to end lives prematurely. Studies such as the present one may contribute towards actively ensuring the subject is placed on the agenda of specialist conferences and symposiums.

## Data Availability

The datasets used and/or analyzed as part of the present study are available from the corresponding author on reasonable request.

## Abbreviations

EURELD: European end-of-life decision
GP: General practitioners
KoR: Killing on request
PAS: Physician-assisted suicide
